# Evaluation of the relationship between cranial magnetic resonance imaging findings and clinical status in children with cerebral palsy

**DOI:** 10.3906/sag-2010-187

**Published:** 2021-06-28

**Authors:** Nihan ŞIK, Fatma Ceren SARIOĞLU, Özgür ÖZTEKİN, Berrak SARIOĞLU

**Affiliations:** 1 Division of Pediatric Emergency Care, Department of Pediatrics, Dokuz Eylül University, Faculty of Medicine, İzmir Turkey; 2 Division of Pediatric Radiology, Department of Radiology, Dokuz Eylül University Faculty of Medicine, İzmir Turkey; 3 Department of Radiology, Health Sciences University, İzmir Tepecik Training and Research Hospital, İzmir Turkey; 4 Division of Pediatric Neurology, Department of Pediatrics, İzmir Tepecik Training and Research Hospital, İzmir Turkey

**Keywords:** Cerebral palsy, comorbidity, gross motor function, cranial MRI

## Abstract

**Background/aim:**

The objective of this study was to evaluate the relationship between cranial magnetic resonance imaging (MRI) findings and clinical features in cerebral palsy (CP).

**Materials and methods:**

Children aged 3 to 18 years, who were followed with the diagnosis of CP between January 2012 and September 2015, were included. The type of CP was classified using the European Cerebral Palsy Monitoring Group’s classification system and then, patients were divided into two groups as spastic or nonspastic groups. The Gross Motor Function Classification System (GMFCS) was used to determine the level of mobility. According to the GMFCS, levels 1, 2, and 3 were grouped as mobile, and levels 4 and 5 were grouped as immobile. Cranial MRI findings were reevaluated by a voluntarily radiologist and grouped as periventricular leukomalacia (PVL) (grades 1, 2, and 3), cerebral atrophy, migration anomaly, cerebellar involvement, basal ganglion involvement, and normal MRI findings.

**Results:**

Sixty-two patients were enrolled. The rate of mobile patients did not differ between the spastic and nonspastic groups. The incidence of PVL was significantly higher in cases of prematurity and spastic CP ( *p* < 0.05). The rate of mobilization was significantly lower and the rate of epilepsy was significantly higher in patients with PVL. Immobile patients were more common among cases of grade 3 PVL ( *p* < 0.05).

**Conclusion:**

The most common cranial MRI pathology was PVL, and the presence of PVL and its grade might help clinically assess the patient’s CP type and level of mobilization. While pathology was observed mostly in cranial MRI in cases of CP with similar clinical features, the fact that cranial MRI was completely normal for 14.5% of the cases suggests that there may be some pathologies that we could not identify with today’s imaging technology.

## 1. Introduction

Cerebral palsy (CP) is the term for a group of permanent but nonprogressive disorders of movement or posture due to an early insult to the developing immature brain in the prenatal, natal, or early postnatal periods [1]. It is the most common cause of childhood disability, and despite increases in medical interventions, the occurrence of CP remains stable at a frequency of 1–2/1000 live births [2–5]. Early diagnosis is important for an expeditious etiological assessment, to provide early intervention, and to prevent complications [6]. Neuroimaging plays an important role for the evaluation of the site, timing, and severity of the brain injury, so cranial magnetic resonance imaging (MRI) has been recommended by the American Academy of Neurology since 2004. Studies reported that neuroimaging patterns also correlated with the subtype of the disease and clinical findings such as motor function [4]. The European Cerebral Palsy Study reported that abnormal cranial MRI findings were found in 88.3% of patients [7]. The clinical severity is related to motor function, which is crucial for the coordination of adequate therapy and rehabilitation. The most commonly used classification system to group the severity of motor impairment of patients with CP is the Gross Motor Function Classification System (GMFCS), which divides motor function into five groups based on walking, sitting, and wheeled mobility [8]. The objective of this study was to evaluate the relationship between cranial MRI findings and clinical features in children with CP between 3 and 18 years of age.

## 2. Materials and methods

This was a retrospective chart review performed in the pediatric neurology clinics of the Katip Çelebi University Faculty of Medicine and İzmir Tepecik Training and Research Hospital. The study protocol was approved by the Ethics Committee of İzmir Tepecik Training and Research Hospital (approval number: 24.11.2015 **/** 1). Children aged 3 to 18 years, who were followed with the diagnosis of CP between January 1, 2012 and September 1, 2015, were included. We used International Classification of Diseases codes for CP to identify patients. We obtained information from a computer database, electronic medical records, and medical charts. Patients with genetic, metabolic, or neurodegenerative disorders and insufficient data were excluded. Demographics and birth characteristics, etiological causes, history of hospitalization, need for mechanical ventilation, age at diagnosis, neurological examination findings, and comorbidities were recorded. The type of CP was classified as spastic (tetraplegic, diplegic, or hemiplegic), dyskinetic (dystonic or choreoathetoid), ataxic, or mixed type using the European Cerebral Palsy Monitoring Group’s classification system [9]. The GMFCS was used to determine the level of mobility among 5 possible levels [8]. According to the GMFCS, levels 1, 2, and 3 were grouped as mobile, and levels 4 and 5 were grouped as immobile. Cranial MRI findings were reevaluated by a radiologist who voluntarily participated in our study and were classified as periventricular leukomalacia (PVL) (grades 1, 2, and 3), cerebral atrophy, migration anomaly, cerebellar involvement, basal ganglion involvement, and normal MRI findings. Neurological examination findings were simultaneously recorded at the time of performing cranial MRI.

Statistical analyses were performed using SPSS software version 22.0 (IBM Cop., Armonk, NY, USA) for Windows. Categorical and continuous variables were reported as frequencies and percentiles, means with standard deviations (SDs), or medians with interquartile ranges (IQRs). The Mann–Whitney *U* test was used to compare nonparametric variables and Student’s t test was used for parametric data. Comparisons of qualitative variables between groups were performed with chi-square and Fisher’s exact tests. Values of *p* < 0.05 were considered statistically significant.

## 3. Results

Sixty-two patients were enrolled in the study. The mean age was 9.31 ± 4.13 years. Of the patients, 42 (67.7%) were male. Eighteen (29.1%) patients were born before the 37th gestational week and 44 (70.1%) patients were born at term. The mean birth weight was 2629 ± 90 g. Thirty-two (51.6%) of the patients were born by normal spontaneous vaginal delivery and 30 (48.4%) were born by cesarean section. Perinatal asphyxia was detected in 37 cases (59.7%), postnatal infection in 2 cases (3.2%), kernicterus in 4 cases (6.5%), and multiple pregnancy in 4 cases (6.5%). Thirty-three (61.3%) patients had a history of hospital stays. There was a history of need for mechanical ventilation in 19 cases (30.6%). The median age at diagnosis of CP was 3.08 years (IQR: 2.0–5.0). The median age at starting special education was 3.04 years (IQR: 1.0–6.0) as shown in Table 1. 

**Table 1:** Demographics, etiological factors, GMFCS levels, and comorbidities of the patients in the study.

Variables (n: 62)	
Sex, n (%)	
Female	20 (32.3% )
Male	42 (67.7% )
Age in years, mean ± SD (min–max)	9.31 ± 4.13 (4.0–17.0)
Born at, n (%)	
<37th gestational week	18 (29%)
Full term	44 (71%)
Birth weight (g), mean±SD (min-max)	2629 ± 90 (700–5000)
Birth method, n (%)	
Normal spontaneous vaginal delivery	32 (51.6%)
Cesarean	30 (48.4%)
Perinatal asphyxia, n (%)	37 (59.7%)
Postnatal infection, n (%)	2 (3.2%)
Kernicterus, n (%)	4 (6.5%)
Multiple pregnancy, n (%)	4 (6.5%)
History of stay at hospital, n (%)	38 (61.3%)
History of mechanic ventilation n (%)	19 (30.6%)
Age at diagnosis (years), median (IQR)	3.08 (2.0–5.0)
GMFCS levels, n (%)	
Level 1	-
Level 2	15 (24.2% )
Level 3	18 (29.0% )
Level 4	11 (17.7% )
Level 5	18 (29.0% )
Comorbidities, n (%)	
Hearing impairment	9 (14.5%)
Visual impairment	19 (30.6%)
Mental retardation	50 (80.6%)
Speech impairment	44 (70.9%)
Epilepsy	26 (41.9%)

GMFCS: Gross motor function classification system.

Fifty (80.6%) of our cases were spastic, 3 (4.8%) were ataxic, 4 (6.4%) were dyskinetic, and 5 (8%) were the mixed type of CP. Of the patients with spastic CP, 24 (38.7%) of them were tetraplegic, 15 (24.2%) were diplegic, and 11 (17.7%) were hemiplegic. Of those with dyskinetic CP, 3 (4.8%) of them had dystonic and 1 (1.8%) had choreoathetoid CP, as shown in Table 2. There was no difference in hospitalization or need for mechanic ventilation between patients with spastic and nonspastic CP. 

**Table 2:** Types of CP of the patients in the study.

Type of CP		n (%)
Spastic	Tetraplegia	24 (38.7%)
	Diplegia	15 (24.2%)
	Hemiplegia	11 (17.7%)
Dyskinetic	Dystonic	3 (4.8%)
	Choreoathetoid	1 (1.6%)
Ataxic		3 (4.8%)
Mixed		5 (8.1%)

CP: Cerebral palsy.

According to the GMFCS, there was no patient at level 1. Fifteen (24.2%) patients were at level 2, 18 (29.0%) were level 3, 11 (17.7%) were level 4, and 18 (29.0%) were level 5 (Table 1). We grouped the cases at GMFCS levels of 1, 2, and 3 as the mobile group (n: 33, 53.2%) and those at levels 4 and 5 (n: 29, 46.8%) as immobile. There was no difference in hospitalization or need for mechanic ventilation between the mobile and immobile groups. In addition, the rate of mobile patients did not differ between the spastic and non-spastic groups.

Fifty-three (85.4%) patients had comorbidities, 50 (80.6%) had mental retardation, 44 (70.9%) had speech impairment, 26 (41.9%) had epilepsy, 19 (30.6%) had visual impairment, and 9 (14.5%) had hearing impairment (Table 1). Speech impairment was detected in 16 (48.4%) of the mobile and 28 (96.5%) of the immobile patients; the rate of speech impairment was significantly higher in the immobile group ( *p* : 0.001). Visual impairment was detected in 6 (18.1%) of the mobile and 13 (44.8%) of the immobile patients; the rate of visual impairment was also higher among immobile patients ( *p* : 0.023). There was no difference in rates of epilepsy, hearing impairment, or intellectual disability between mobile and immobile patients. 

Cranial MRI findings were as follows: periventricular leukomalacia in 33 cases (53.2%), cerebral atrophy in 8 cases (12.9%), migration anomaly in 6 cases (9.7%), cerebellar involvement in 2 cases (3.2%), basal ganglion involvement in 4 cases (6.4%), and normal findings in 9 cases (14.5%), as shown in Table 3. The most common finding was PVL. Of the 33 cases of PVL, 5 (15.2%) were grade 1, 5 (15.2%) were grade 2, and 23 (69.7%) were grade 3. PVL was detected in 13 (72.2%) of 18 patients who were born preterm and in 20 (45.4%) of 45 patients who were born at term; the incidence of PVL was thus significantly higher in cases of prematurity ( *p* : 0.045). There was no difference in hospitalization or need for mechanic ventilation between patients with or without PVL. Among the 33 patients with PVL, 20 (60.6%) of them had epilepsy, while among the 29 patients without PVL, 6 (20.6%) had epilepsy; the rate of epilepsy was significantly higher in patients with PVL ( *p* : 0.008). There was no difference in other comorbidities between patients with or without PVL. According to the GMFCS groups, 12 patients (36.3%) with PVL and 21 (72.4%) patients without PVL were mobile; the rate of mobilization was significantly lower in patients with PVL ( *p* : 0.005). When we compared patients with spastic and non-spastic CP according to the presence of PVL, 30 (60.0%) of 50 patients in the spastic group and 3 (25.0%) of 12 patients in the non-spastic group had PVL; the rate of PVL was higher in patients with spastic CP ( *p* : 0.031). We evaluated PVL grades and GMFCS levels; in the mobile group, 3 of 12 patients (25.0%) were PVL grade 1, 4 (33.3%) were grade 2, and 5 (41.6%) were grade 3, and in the immobile group, 2 of 21 (9.5%) patients were grade 1, 1 (4.7%) was grade 2, and 18 (85.7%) were grade 3. Immobile patients were more common in PVL grade 3 ( *p* : 0.042). Subtypes of the children with normal cranial MRI were as follows: 4 of them were spastic diplegic, 2 were dyskinetic, 2 were ataxic, and 1 had the mixed type of CP.

**Table 3:** Cranial MRI findings of the patients in the study.

Cranial MRI findings	PVL	Cerebralatrophy	Migration anomaly	Cerebellar involvement	Basal ganglion involvement	Normal
n (%)	33 (53.2%)	8 (12.9%)	6 (9.7%)	2 (3.2%)	4 (6.4%)	9 (14.5%)

MRI: Magnetic resonance imaging, PVL: Periventricular leucomalasia.

## 4. Discussion

Male dominance was reported in previous studies on CP, as the male/female ratio was 1.4 in the United States and as 2.0 in the Netherlands [10]. In our study, it was 2.0, in accordance with the literature. Of the etiological factors, prematurity ranks first place in developed countries, but in developing countries with poor birth conditions, kernicterus, perinatal asphyxia, and postnatal infections are dominant [11]. In our study, the most common etiological factor was perinatal asphyxia, followed by prematurity. Von Wendt et al. found no etiological factor in 13.0% of cases of CP [12]. Likewise, 24.1% of our patients had no known etiological factor. 

Spastic CP is the most common type. Spastic tetraplegia is more common in developing countries, but in developed countries, in parallel to the increasing survival rates in cases of prematurity, spastic diplegia is more frequent [13]. In our study, tetraplegia was the most common subtype. Mental retardation is seen to varying degrees, and associated with the type of CP [11]. While cortical grey matter is usually preserved in spastic diplegia, cognitive functions are slightly affected, and it was reported that approximately half of the patients with spastic diplegia had normal intelligence quotients. In spastic tetraplegia, however, mental retardation is more common and severe [14]. In our study, mental retardation was detected in 80.6% of our patients, and we think that this high frequency was related to the high rate of patients with spastic CP in our study population. Epilepsy was seen in 41.4%-89.9% of patients and was more common in cases of spastic tetraplegia [14,15]. Likewise, 41.9% of our patients had epilepsy and patients with spastic tetraplegia had an epilepsy rate of 58.3%, in accordance with the literature.

There are no specific criteria to group the severity of the movement disorders. The GMFCS, which was proposed by Palisano et al. in 1997 and revised in 2007, is easy to use and does not require special training, and the use of this classification system has become more common. It is a good predictor for motor prognosis and can help physicians consider the development of orthopedic problems and plan treatment strategies [8,11]. In a review, the majority of the patients were at level 1 (27.3%) and 65.5% of the population was at level 1 or 2 [16]. In our study, there were no patients at level 1 and 24.2% were at level 2, our rates being lower than those in the literature. Additional health problems that may accompany CP are thought to be related to less frequent hospital admissions of these patients. In addition, the frequency of comorbidities varied according to the GMFCS levels. It was reported that as the GMFCS level increased, the accompanying disorders and their severity were increased, and comorbidities were associated with CP type and GMFCS levels [17]. However, we found few pertinent studies in the literature. In our study, speech and visual impairments were significantly higher in the immobile group according to the GMFCS levels, but there was no difference for epilepsy, mental retardation, or hearing impairment.

Cranial MRI is the most recommended neuroimaging modality. The most common pathologies reported in the literature are white/grey matter lesions and cerebral malformations. In the study of the European Cerebral Palsy Group, abnormal cranial MRI findings were detected in 88.3% of the patients. The most common findings were PVL (42.5%), basal ganglion involvement (12.8%), and cortical/subcortical damage (9.4%) [4]. Ali et al. evaluated 1100 cases of CP and found that the most common pathology was cerebral atrophy, followed by PVL [18]. Hayakawa et al. evaluated children with spastic CP and reported that all of them had abnormal cranial MRI findings, and the most common finding was PVL [19]. Kulak et al. reported PVL to be the most common cranial MRI finding in cases of spastic diplegia rather than tetraplegia or hemiplegia [20]. In our study, 85.5% of the patients had abnormal cranial MRI findings; the most common pathology was PVL, followed by cerebral atrophy. PVL was more common in patients with spastic CP, and the majority had the spastic tetraplegic subtype. The rate of spastic tetraplegia cases with PVL was higher than expected, which may be related to the majority of the study population consisting of patients with moderate to severe PVL. Shang et al. reported that comorbidities were more common in cases of spastic CP and in PVL, and the most common comorbidities were epilepsy and hearing and visual impairment [21]. In our study, epilepsy was more common in patients with PVL but other comorbidities did not differ between patients with and without PVL. In a review, grey matter lesions were found to be associated with GMFCS levels of 4 and 5, representing immobile patients [22]. In another trial, the most common lesion was detected as PVL, but approximately two-thirds of those patients were at GMFCS level 1 or 2 while the majority of patients with grey matter lesions were classified as levels 3-5 [16]. In contrast, 36.3% of our patients with PVL were at GMFCS level 1, 2, or 3. The presence of PVL was considered to have a negative effect on gross motor function. There are few studies evaluating PVL grades and clinical features. Shang et al. reported an association between clinical severity and PVL grades; there were no spastic tetraplegic patients at PVL grade 1, but at PVL grade 3, 56.3% of them were tetraplegic [21]. In accordance with the literature, 73.9% of our PVL grade 3 patients had the spastic tetraplegic CP subtype. In addition, 85.7% of immobile patients were PVL grade 3. The PVL grades of spastic tetraplegic and immobile cases were observed to be more severe.

Normal cranial MRI findings were observed in 9 (14.5%) of our patients. The rate was reported as 29.0%, and 10.4% and it was more common in patients with spastic diplegia [16,23,24]. Likewise, nearly half of our patients with normal neuroimaging were spastic diplegic. In addition, patients with normal MRI findings were more likely to have no perinatal adversity in previous studies [23,24]. 

There were some limitations related to the retrospective nature of our study; missing data may lead to the underestimation of the real number of cases. Furthermore, the sample size was relatively small due to the low prevalence of the disease.

We conclude that the most common cranial MRI pathology was PVL, and the presence of PVL and its grade might help clinically assess the patient’s CP type and level of mobilization. While pathology was observed mostly in cranial MRI in cases of CP with similar clinical features, the fact that cranial MRI in other cases was completely normal suggests that there may be some pathologies that we could not identify with today’s imaging technology. It is thought that the number of such pathologies will decrease gradually with the development of molecular imaging methods in the future.

## Funding

This research did not receive any specific grant from funding agencies in the public, commercial, or not-for-profit sectors. 
